# Traffic-related pollution and asthma prevalence in children. Quantification of associations with nitrogen dioxide

**DOI:** 10.1007/s11869-014-0265-8

**Published:** 2014-05-10

**Authors:** Graziella Favarato, H. Ross Anderson, Richard Atkinson, Gary Fuller, Inga Mills, Heather Walton

**Affiliations:** 1 Respiratory Epidemiology, Occupational Medicine and Public Health and MRC-PHE Centre for Environment and Health, Imperial College, London, London, UK; 2 MRC-PHE Centre for Environment and Health, King’s College London, London, UK; 3 MRC-PHE Centre for Environment and Health, Population Health Research Institute, St George’s, University of London, London, UK; 4 Public Health England, Centre for Radiation, Chemical and Environmental Hazards, London, UK; 5 NIHR BRC at Guy’s & St Thomas’ NHS Foundation Trust and King’s College London, MRC-PHE Centre for Environment and Health, King’s College London, London, UK

**Keywords:** Air Pollution, Asthma prevalence, Traffic, Meta-analysis, Review

## Abstract

Ambient nitrogen dioxide is a widely available measure of traffic-related air pollution and is inconsistently associated with the prevalence of asthma symptoms in children. The use of this relationship to evaluate the health impact of policies affecting traffic management and traffic emissions is limited by the lack of a concentration-response function based on systematic review and meta-analysis of relevant studies. Using systematic methods, we identified papers containing quantitative estimates for nitrogen dioxide and the 12 month period prevalence of asthma symptoms in children in which the exposure contrast was within-community and dominated by traffic pollution. One estimate was selected from each study according to an a priori algorithm. Odds ratios were standardised to 10 μg/m^3^ and summary estimates were obtained using random- and fixed-effects estimates. Eighteen studies were identified. Concentrations of nitrogen dioxide were estimated for the home address (12) and/or school (8) using a range of methods; land use regression (6), study monitors (6), dispersion modelling (4) and interpolation (2). Fourteen studies showed positive associations but only two associations were statistically significant at the 5 % level. There was moderate heterogeneity (I^2^ = 32.8 %) and the random-effects estimate for the odds ratio was 1.06 (95 % CI 1.00 to 1.11). There was no evidence of small study bias. Individual studies tended to have only weak positive associations between nitrogen dioxide and asthma prevalence but the summary estimate bordered on statistical significance at the 5 % level. Although small, the potential impact on asthma prevalence could be considerable because of the high level of baseline prevalence in many cities. Whether the association is causal or indicates the effects of a correlated pollutant or other confounders, the estimate obtained by the meta-analysis would be appropriate for estimating impacts of traffic pollution on asthma prevalence.

**Electronic supplementary material:**

The online version of this article (doi:10.1007/s11869-014-0265-8) contains supplementary material, which is available to authorized users.

## Background

Ambient nitrogen dioxide (NO_2_) has been associated with mortality and a range of morbidity outcomes (US EPA 2008; WHO 2006). Until recently, these associations were considered more likely to be explained by toxicants associated with NO_2_ than by NO_2_ per se. However, the most recent reviews by these same authorities have shifted their opinion to one in which NO_2_ is deemed to play a direct causal role, at least in part (US EPA 2013; WHO 2013). Irrespective of its causal role, nitrogen dioxide remains the most widely available pollutant measure of proximity to traffic emissions and hence the most widely reported in epidemiological studies. The relative specificity of nitrogen dioxide as a marker of traffic proximity makes it a suitable and convenient metric for modelling the health impacts of traffic pollution and evaluating abatement policies.

Asthma is a common chronic disease in childhood (Lai et al. 2009) and globally accounts for an estimated 7 % of disability life-years among the 5 to 14-year-age group (Institute of Health Metrics and Evaluation 2013). A number of studies have observed associations between the incidence and/or prevalence of asthma and variations in long-term exposure to nitrogen dioxide within urban environments in which traffic emissions are the main source of pollution (Health Effects Institute 2010). These associations are frequently observed at levels below current WHO guidelines and show little evidence of a threshold (WHO 2006, 2013).

The purpose of the current review of published evidence is to develop concentration-response functions for NO_2_ and asthma that would be suitable for quantifying the impacts of traffic and traffic policies on asthma in situations where fine spatial scale models of NO_2_ concentrations are available. In a previous meta-analysis designed to investigate the role of air pollution in the onset of asthma, we obtained a significant increase in the relative risk of asthma incidence associated with NO_2_ (13 studies, combined odds ratio 1.07, 95 % CI 1.02 to 1.13) (Anderson et al. 2013). However, this estimate is not suitable for health impact assessment because the incidence of asthma is a poor reflection of the current burden (Strachan et al. 1996) and baseline rates for incidence are rarely available. A better reflection of burden is provided by the 12-month period prevalence metric, and since this is commonly used for national health surveys and ad hoc epidemiological cross-sectional studies of asthma, baseline rates are more widely available. To date, impact assessments of NO_2_ and asthma have been based on concentration-response functions from single studies (Perez et al. 2009). There are, however, good arguments for basing such impact assessments on a meta-analysis of all published evidence. Our paper addresses this gap by reporting the results of a systematic review and meta-analysis aimed at the development of a concentration-response function for NO_2_ and the prevalence of asthma symptoms that is appropriate for assessing the health impact of traffic policies.

## Methods

The aim of the literature search was to identify within-community population based studies with estimates that quantified in continuous form (i.e. per unit of air pollution) associations between nitrogen dioxide and the prevalence of asthma, defined as period prevalence (12 months) measures of either asthma symptoms (wheeze) or asthma diagnosis. The search string is detailed in Online Resource 1 along with the PRISMA flow diagrams (Moher et al. 2009). We searched Medline, Embase and ISI Web of Science up to 1 March 2013. Following sifts by title and abstract, the full text of potentially eligible articles was assessed by two reviewers (GF and HRA). Relevant details were extracted into a relational database (Microsoft ACCESS version 2002, Microsoft Corporation, Redmond, WA, USA) of data from epidemiological studies of chronic exposure to outdoor air pollution and respiratory outcomes (Air Pollution Epidemiology Database—APED). The protocols for the APED database have been fully described elsewhere (Anderson et al. 2013). In brief, the database comprised two levels, the first relating to the study and the second to individual estimates from that study. From this database, we identified those studies meeting the criteria for this specific analysis. Details on exposure and respiratory outcomes were entered exactly as described in the results section of each paper. The odds ratios were standardised to 10 μg/m^3^. Potential confounding factors considered by each study were classified by APED into the following five categories: (1) *indoor*—gas stoves, pets, damp etc.; (2) *socioeconomic*—occupation, education etc.; (3) *tobacco smoke*—current parental, in utero; (4) *demographic*—age, sex, ethnicity etc.; and (5) *other*—breast feeding, parental allergies, past respiratory infections etc.

For the current meta-analysis, we selected only one measure of prevalence per study. Where prevalence was reported by a cohort study, the estimate from the most recent follow-up was selected. If a study reported more than one measure of asthma prevalence, we selected one estimate according to the following priority: (1) wheeze period prevalence and (2) asthma diagnosis period prevalence. The period prevalence selected was the conventional one of 12 months prior to interview. The period of exposure differed from study to study but we chose that which was most concurrent with the assessment of asthma symptoms. If only estimates for nitrogen oxide (NO_*x*_) concentrations were reported, these were scaled to nitrogen dioxide using a factor of 0.44 based on the ratio that fell midway between the average ratios for roadside and urban background monitoring sites in London for 2001, as previously described (Anderson et al. 2013). We calculated summary effects estimates using random- and fixed-effects models (DerSimonian and Laird 1986), and heterogeneity using the *I* ^*2*^ statistic which indicates the proportion of variability between effect estimates due to heterogeneity (Higgins and Thompson 2002). We investigated publication (small study) bias visually with funnel plots (Sterne et al. 2000) and two statistical tests (Begg and Mazumdar 1994; Egger et al. 1997). The *metan*, *metafunnel and metabias* commands in STATA version 10.1 were used (Stata Corporation, College Station, TX, USA). Where numbers permitted, we carried out sensitivity analyses to evaluate the algorithm for outcome selection and to compare estimates according to the method of exposure assessment.

## Results

The original search for papers of chronic exposure to air pollution and respiratory outcomes identified 6,906 possibilities from which 334 were eligible for the APED database. From these, 334 papers, 20 based on 18 individual studies, met the criteria for inclusion in the meta-analysis of NO_2_/NO_x_ and asthma prevalence (Esplugues et al. 2011; Gauderman et al. 2005; Gehring et al. 2010; Gruzieva et al. 2013; Hirsch et al. 1999; Janssen et al. 2003; Kim et al. 2004, 2011; Kramer et al. 2000, 2009; Mi et al. 2006; Morgenstern et al. 2008; Oftedal et al. 2009; Penard-Morand et al. 2010; Pikhart et al. 2000; Sonnenschein-van der Voort et al. 2012; Svendsen et al. 2012; Zhao et al. 2008). Twelve studies were from Europe, three from Asia and three from the USA. The ages of subjects ranged from 1 to 17 years but the majority (14) included children between the ages of 5 and 12 years. All studies considered potential confounders, and while these varied considerably in their range and detail, the great majority included at least one confounder from each of the following broad categories: *indoor*, *socioeconomic*, *smoking*, *demographic*, and *other* (Table 1). The statistical approach to these confounders varied. Some studies included all the available confounders in the final model while others applied a stepwise approach and excluded those that were not deemed to be important on the basis of arbitrary criteria (e.g. changed the final odds ratio by less than 5 %, were not significant *P* < 0.15) or prior analyses which had found that the variable was not associated with exposure or outcome.

**Table 1:** Details of studies, and measures and standardised estimates for NO_2_ and asthma prevalence selected for meta-analysis

Study	No. of participants and age (year)	Outcome	Exposure assessment: place and method	NO_2_ concentrations mean (range)^a^ μg/m^3^	Odds ratio for NO_2_ standardised to 10 μg/m^3^ (95 % CI)^b^	Confounders considered^c^
Esplugues et al. (2011) Spain	*n* = 352 age = 1	Wheeze: last 12 months	Home address: land use regression model	27.4 (18.4–37.1 IQR)	1.04 (0.85 to 1.27)	1,2,3,4,5
Gauderman et al. (2005) USA	*n* = 208 age 14–17	Wheeze: last 12 months	Home address: study passive samplers	58.9 (24.7–98.5) (between community)	1.64 (1.06 to 2.54)	1,2,3,4,5
Gehring et al. (2010) Netherlands	*n* = 3,863 age 8	Wheeze: last 12 months	Home address: land use regression model	25.4 (12.6–58.4)	1.01 (0.87 to 1.18)	1,2,3,4,5
Gruzieva et al. (2013) Sweden	*n* = 3,633 age 12	Wheeze: last 12 months	Home address: dispersion model	5.2	0.97 (0.89 to 1.07)	1,2,3,4
Hirsch et al. (1999)	*n* = 5,421 age 5–7, 9–11	Wheeze: last 12 months	Home address: interpolation (kriging) based on routine monitoring stations	33.8 (17.1–56.0)	1.13 (0.93 to 1.37)	1,2,3,4,5
Janssen et al. (2003) Netherlands	*n* = 2,071 age 7–12	Wheeze: last 12 months	School address: study monitors	34.8 (26.8–44.4)	1.37 (0.99 to 1.89)	1,2,3,4,5
Kim et al. (2004) USA	*n* = 1,109 age 7–10	Asthma: last 12 months	School address: study passive samplers	44 (36–59)	1.03 (0.96 to 1.11)	1,2,3,5
Kim et al. (2011) S Korea	*n* = 2,365 age 10	Wheeze: last 12 months	School address: study passive samplers	30.7 (16.5–48.6)	1.27 (1.06 to 1.52)	1,3,4
Kramer et al. (2000) Germany	*n* = 317 age 9	Wheeze: last 12 months (diary)	Home address: interpolation from study passive samplers	54.2 (43.0–67.5)	1.70 (0.90 to 3.21)	1,2,3,4,5
Kramer et al. (2009) Germany	*n* = 2,112 age 6	Asthma or asthmatic/obstructive/spastic bronchitis symptoms: since last follow-up	Home address: land use regression model	23.7 (13.6–42.1)	0.61 (0.36 to 1.03)	1,2,3,4,5
Mi et al. (2006) China	*n* = 1,414 age 13	Wheeze: last 12 months	School address: study passive samplers	63 (47–83)	1.00 (0.74 to 1.35)	1,3,4,5
Morgenstern et al. (2008) Germany	*n* = 3,061 age 6	Asthma or asthmatic/obstructive/spastic bronchitis symptoms: last 12 months	Home address: land use regression model	34.6 (16.0–73.7)	1.05 (0.85 to 1.29)	1,2,3,4,5
Oftedal et al. (2009)	*n* = 2,871 age 9–10	Wheeze: last 12 months	Home address: dispersion model	25.2 (1.4–65.1)	1.01 (0.90 to 1.12)	1,2,3,4,5
Penard-Morand et al. (2010) France	*n* = 4,907 age 9–11	Asthma: last 12 months	School address: dispersion model	43.7 (17.8–78.9)	1.19 (0.92 to 1.53)	1,2,3,4,5
Pikhart et al. (2000) Czech Republic	*n* = 3,680 age 7–10	Wheeze: last 12 months	Average of home and school address: land use regression model	35.8 (IQR 27.9–45.3)	1.16 (0.97 to 1.39)	1,2,3,4
Sonnenschein-van der Voort et al. (2012) Netherlands	*n* = 4,634 age 3	Wheeze last 12 months	Home address: dispersion model	36.2 (27.0 55.7)	0.97 (0.72 to 1.31)	1,2,3,4,5
Svendsen et al. (2012) USA	*n* = 4,231 age 9–11	Wheeze last 12 months	Home and school address: land use regression model.	43.8 (35.2–52.3)	0.90 (0.67 to 1.20)	1,2,3,4,5
Zhao et al. (2008) China	*n* = 1,993 age 11–15	Wheeze last 12 months	School address: study passive samplers	52.3 (37.9–65.2)	1.00 (0.83 to 1.20)	1,2,3,4,5

^a^Unless otherwise indicated

^b^Lower 95 % confidence intervals may differ slightly from published figures due to rounding by ACCESS programme

^c^Key for confounders: 1 = Indoor, 2 = Socioeconomic, 3 = Smoking, 4 = Demographic, 5 = Other

From these 20 papers, 39 estimates for NO_2_ or NO_*x*_ and the period prevalence of asthma were extracted into the ACCESS database [see Online Resource 2]. After excluding earlier reports from the same study and applying the “wheeze symptom first” algorithm, we obtained 18 study-specific estimates, 16 for wheeze symptom and two for asthma diagnosis from those that did not report wheeze symptom. Seventeen of these study-specific estimates were for NO_2_ and one was for NO_*x*_ (scaled to NO_2_—see methods). The details of the standardised estimates are summarised in Table 1.

The measurement of NO_2_ was at the home address for 10 studies, the school for 6 studies and an average of home and school for 2 studies. The methods of exposure assessment were study-specific monitors (6), land use regression (6), dispersion models (4) and interpolation from monitors (2).

**Fig. 1:**
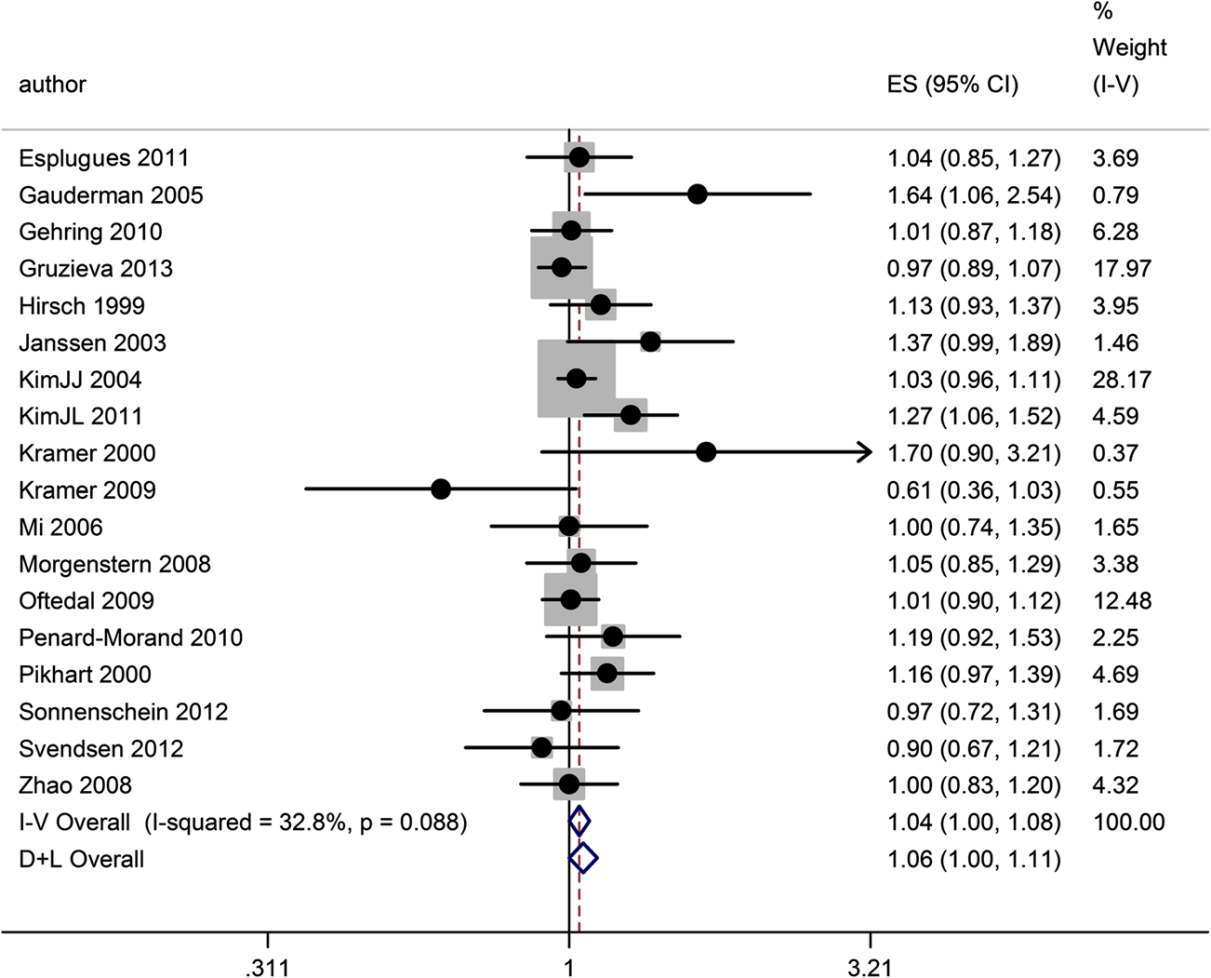
Forest plot and meta-analysis of associations between NO_2_ (per 10 μg/m^3^) and the 12-month period prevalence of asthma symptoms

The results for the meta-analysis of NO_2_ are presented as a forest plot in Fig. 1. There was moderate heterogeneity (*I* ^*2*^ = 32.8 %), and the fixed-effects and random-effects estimates of the odds ratio were, respectively 1.04, 95 % CI 1.00 to 1.08 and 1.06, 95 % CI 1.00 to 1.11. The funnel plot was generally symmetrical (see Online Resource 3) and neither the Begg’s nor Egger’s tests suggested small study bias. A subgroup of 10 studies also reported the 12-month period prevalence of asthma, and the summary estimate for these was very similar to the odds ratio based on all 18 estimates (heterogeneity 0 %, fixed-effects odds ratio (FE-OR) 1.06, 95 % CI 1.01 to 1.12) (data not shown).

**Fig. 2:**
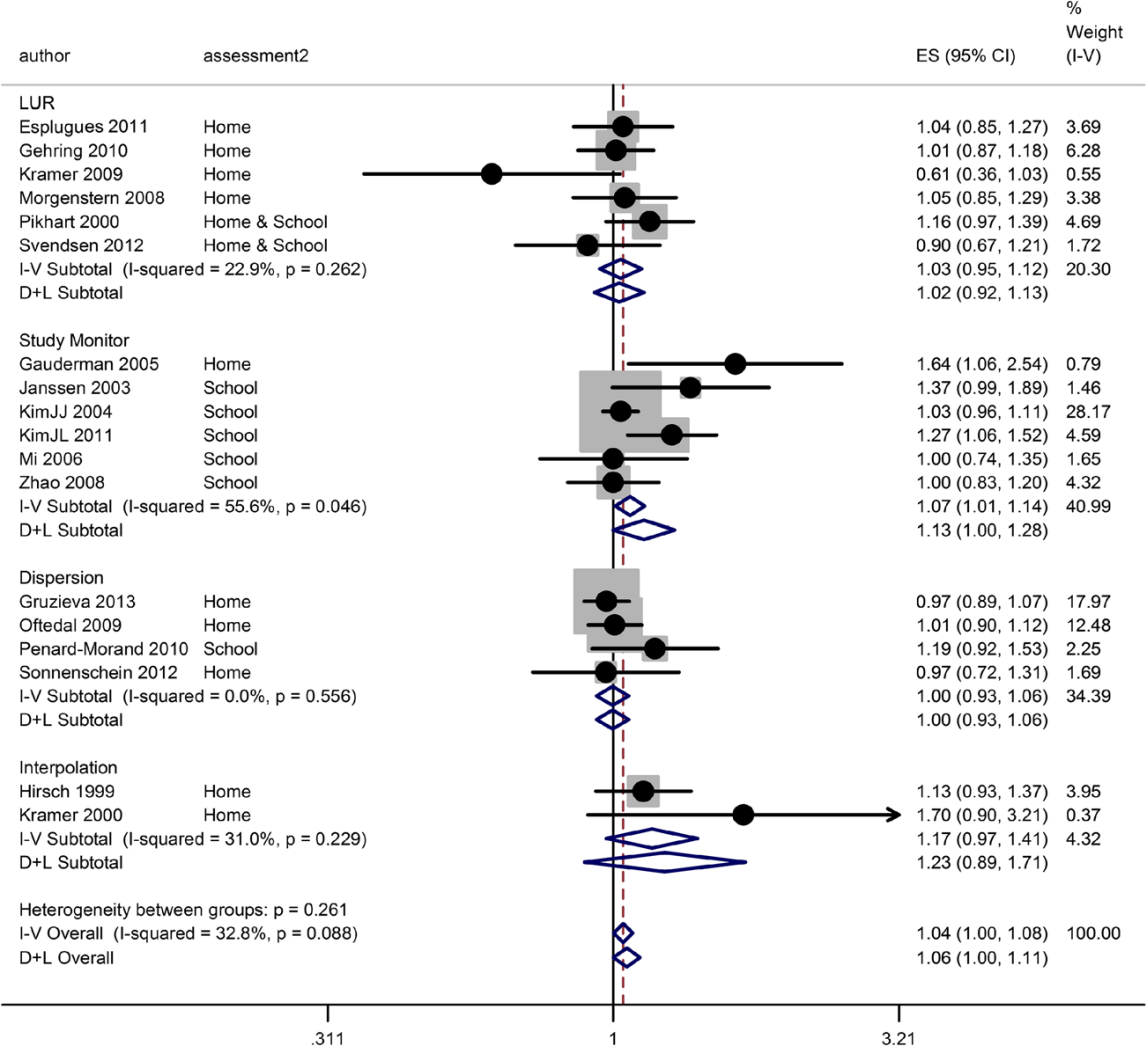
Forest plot and meta-analysis of estimates for NO_2_ (per 10 μg/m^3^) and the 12-month period prevalence of asthma symptoms, stratified by method of exposure assessment

We also investigated whether there was any tendency for estimates to vary by the method of exposure assessment. None of the studies used more than one method, so direct comparison within a study was not possible. The results of the meta-analysis stratified by dispersion model, interpolation, land use regression, or study monitor are shown in Fig. 2. The summary OR estimate for dispersion modelling (four studies) was unity (RE 1.00, 95 % CI 0.93 to 1.06) while that from land use regression was a little larger (RE 1.02, 95 % CI 0.92 to 1.13). The largest estimates were for interpolation (two studies, RE 1.23, 95 % CI 0.89 to 1.71) and study operated monitor (six studies, RE 1.13, 95 % CI 1.00 to 1.28). Among the latter group, five of the six studies measured NO_2_ at the child’s school whereas the other measurement groups were largely based on estimates for the home address. The only study to measure exposure using a study monitor at the home address reported the second largest estimate (OR 1.64, 95 % CI 1.06 to 2.54) (Gauderman et al. 2005). Statistically, there was however no evidence of heterogeneity between the groups, but the power to investigate this was low.

## Discussion

We carried out a meta-analysis of studies reporting associations between nitrogen dioxide and the period prevalence of asthma symptoms or asthma diagnosis. All of the studies were of children but there was considerable heterogeneity in the ages of subjects, the form of the questionnaire and the method of exposure assignment. Among the 18 studies identified, there was moderate heterogeneity and no evidence of publication bias. The random-effects summary odds ratio for 10 μg/m^3^ NO_2_ was 1.06 (95 % CI 1.00 to 1.11).

While there have been numerous reviews of the health effects of traffic pollution, these have mainly focused on the evidence in relation to hazard rather than quantification. The question of quantification places more constraints on study selection because it is necessary to consider not only whether there is evidence of hazard but whether the result of the analysis can be used to quantify health impacts. The latter requires a concentration-response function that links a health outcome for which a baseline can be estimated and an exposure metric that can be replicated by the model being used in the quantification. Thus, estimates that are based on distance from the road or categorical divisions of pollutant concentration were excluded from our review.

The value of meta-analysis is demonstrated by our analysis. The majority (16) of the estimates were not statistically significant at the 5 % level whereas the combined estimate was more precise, having a narrower confidence interval and bordering on statistical significance at the 5 % level. There was a tendency for the larger effects to be based on study monitors, most of which were situated at the child’s school and for the smaller effects to be based on dispersion models, but we did not have sufficient statistical power to confirm this relationship. Furthermore, none of the studies compared different methods of exposure assessment within the same study.

The exhaustive report on traffic and health by the Health Effects Institute (Health Effects Institute 2010) concluded, with respect to asthma incidence and asthma prevalence, that the evidence for associations with NO_2_ was mixed and that the causality of any associations was judged to fall somewhere between “sufficient” and “suggestive but not sufficient” to infer causality. The report regarded the lack of precision in many studies as a major problem but did not attempt a quantitative meta-analysis because of concerns about heterogeneity of methods. We believe that our approach enabled a meta-analysis to be carried out on studies that were sufficiently homogeneous in their methods and we also had the benefit of studies published since the HEI review. The results, along with our previous meta-analysis of incidence (Anderson et al. 2013), contain new studies and strengthen the evidence that there is a small but real association between NO_2_ and increased asthma prevalence in children.

In practice, the period prevalence metric is based on the recollection of asthma symptoms over a prior period, most commonly 12 months. Serious deficiencies in this approach include recall bias, inadequate quantification of the frequency, severity and duration of episodes and an inability to distinguish different phenotypes. A further problem is the lack of standardisation of the questionnaires among the studies included in this review. Notwithstanding these problems, the 12-month period prevalence metric remains the only accepted method that can be applied easily in large populations and can provide sufficient estimates for meta-analysis. Conceptually, the period prevalence of asthma is the net effect of a number of different processes: incidence, prognosis and severity, but the relative importance of these three parameters is not quantifiable at present. In our previous meta-analysis of NO_2_ and the incidence of asthma, based on 13 cohorts (Anderson et al. 2013), we obtained a summary estimate of 1.07 (95 % CI 1.02 to 1.13). This is consistent with at least some of the increased prevalence observed in the present study being explained by an increase in incidence. It is not however clear whether such an increase in incidence might be explained by new cases of asthma (which would not have occurred in the absence of pollution) or by air pollution merely advancing in time the clinical appearance of previously subclinical asthma. Evidence from time-series and panel studies shows convincing short-term associations between air pollution and exacerbation of asthma; this mechanism could explain both the bringing forward of “new asthma” and an increase in prevalence due to an increase in severity.

Causal inference depends first on the degree of confidence that the association is not caused by confounding and second on the plausibility of a direct toxic effect. It is not possible to exclude confounding except to note that all of the studies took a varying but generally wide range of potential confounding factors into account. Confounding by correlated traffic pollutants such as black carbon could not be examined using meta-analysis because few studies reported this metric (see below) or attempted multi-pollutant analyses. In respect of plausibility, the accumulating evidence from controlled animal and human toxicology studies is pointing towards the possibility of a causal role for NO_2_ (at least in part) at ambient concentrations experienced in near traffic environments. Potential mechanisms by which NO_2_ (and other air pollutants) could affect asthma, include: (i) oxidative stress/antioxidant depletion, (ii) increased inflammation and airway hyperresponsiveness, (iii) structural changes in the airways leading to asthma, (iv) enhanced response to allergens and (v) impacts on immunity (Kelly and Fussell 2006; Gowers et al. 2012; US EPA 2013; WHO 2013).

The other measured pollutant which is a good marker of traffic exposure is black carbon particulate matter. Recent reviews have emphasised the importance of this metric but remain uncommitted as to whether it is an indicator or a toxic component of the emissions mixture (Janssen et al. 2011; WHO 2013). Only six studies reported measures of black carbon, all of which used methods of light reflectance or absorbance. We attempted to convert these to a common metric for meta-analysis (mass of elemental carbon) but finally concluded that this involved too many assumptions and too few estimates to merit further analysis. There were four estimates of PM_2.5_ and one of PM_10_, obtained by a range of techniques. All but one of the four studies reported positive associations with asthma prevalence but no individual estimate was significantly positive at the 5 % level. Because of the lack of specificity of PM_2.5_ for primary traffic emissions and the small number of estimates available, we did not take the analysis further.

One of the policy drivers behind our study was the need to improve our tools for estimating impacts of traffic pollution and evaluating measured or modelled changes in health predicted from control measures such as congestion charging schemes or low emissions zones. Because the studies reviewed here employed address- or school-based exposure assignment, estimates based on these studies are suitable for application to small-scale spatial models such as those being increasingly deployed to support air quality management in many cities. We were confined to single pollutant models because too few studies reported multi-pollutant models for meta-analysis. However, if NO_2_ is being used as the most widely available marker for correlated traffic pollutants as well as for any direct effects it may have, a single pollutant model should suffice. Further, a meta-analytic estimate based on single pollutant models derived from a larger number of studies has the theoretical advantage of greater transferability. A caveat to this statement is that our meta-analysis combines estimates from locations that vary by traffic density, fleet composition and time periods when primary NO_2_ emissions were rising in Europe. Further study is required to assess the degree of effect modification these factors have on the NO_2_ association.

Although we observed only moderate heterogeneity in our meta-analysis, it is also likely that the role of air pollution in asthma prevalence will vary from place to place because the mix of other aetiological and environmental factors for asthma and of air pollutants is also likely to vary. For these reasons, the application of concentration-response functions such as we have obtained is most suitable for evaluating the marginal impacts of increments in pollution rather than absolute levels of burden.

Since the cut-off date for our study, the results of the European Study of Cohorts for Air Pollution Effects (ESCAPE) study for traffic pollution and asthma prevalence have been presented in abstract form (Mölter et al. 2013) and the full report is being considered for publication. These results are based on five cohort studies of which earlier reports for four are already represented in the present analysis. Using a fresh and standardised approach to estimating exposure at the address of the cohort subjects (land use regression), ESCAPE reported an estimate of the odds ratio for a 10 μg/m^3^ increment in NO_2_ of 1.12, 95 % CI 0.82 to 1.51, which, while twice as high as our estimate, displays poor precision The pollutants investigated by ESCAPE also included NO_*x*_, PM_10_, PM_2.5_, Coarse PM, and PM_2.5_ absorbance and none of these were significantly associated with asthma prevalence whether the pollution assessment was at birth or later in childhood. There did not appear to be any measurable difference between those pollutants that are more specific for traffic (NO_*x*_, NO_2_, PM_2.5_ absorbance) and pollutants with a wider distribution such as PM_2.5_. It seems unlikely that incorporation of the newer results from ESCAPE would materially change the results of the current meta-analysis.

In summary, using systematic review and meta-analysis, we have strengthened the evidence for an association between NO_2_ and asthma among within-community studies in which the exposure contrast is due to traffic proximity. The results suggest that NO_2_ or correlated pollutants may make a small proportional contribution to asthma prevalence in children. The relative contributions of incidence, prognosis and severity to this effect remain unclear. Our estimate is suitable for quantification of asthma burden in situations where estimates of the asthma prevalence baseline can be made and fine spatial scale models of nitrogen dioxide are available.

## Electronic supplementary material

Below is the link to the electronic supplementary material.

Online Resource 1 (PDF 87 kb)

Online Resource 2 (PDF 60 kb)

Online Resource 3 (PDF 50 kb)
