# Supplementary material for: Traffic-related pollution and asthma prevalence in children. Quantification of associations with nitrogen dioxide
Source: Air Qual Atmos Health. 2014 May 10;7(4):459–66. doi: 10.1007/s11869-014-0265-8 (PMC4239711; doi:10.1007/s11869-014-0265-8)
Supplement: Supplementary file 1 — (PDF 87 kb) [file 11869_2014_265_MOESM1_ESM.pdf]

Supplementary material for *Traffic-related pollution and asthma prevalence in children. Quantification of associations with nitrogen dioxide*.

Online Resource 1.

PRISMA Flow diagram Stage 1

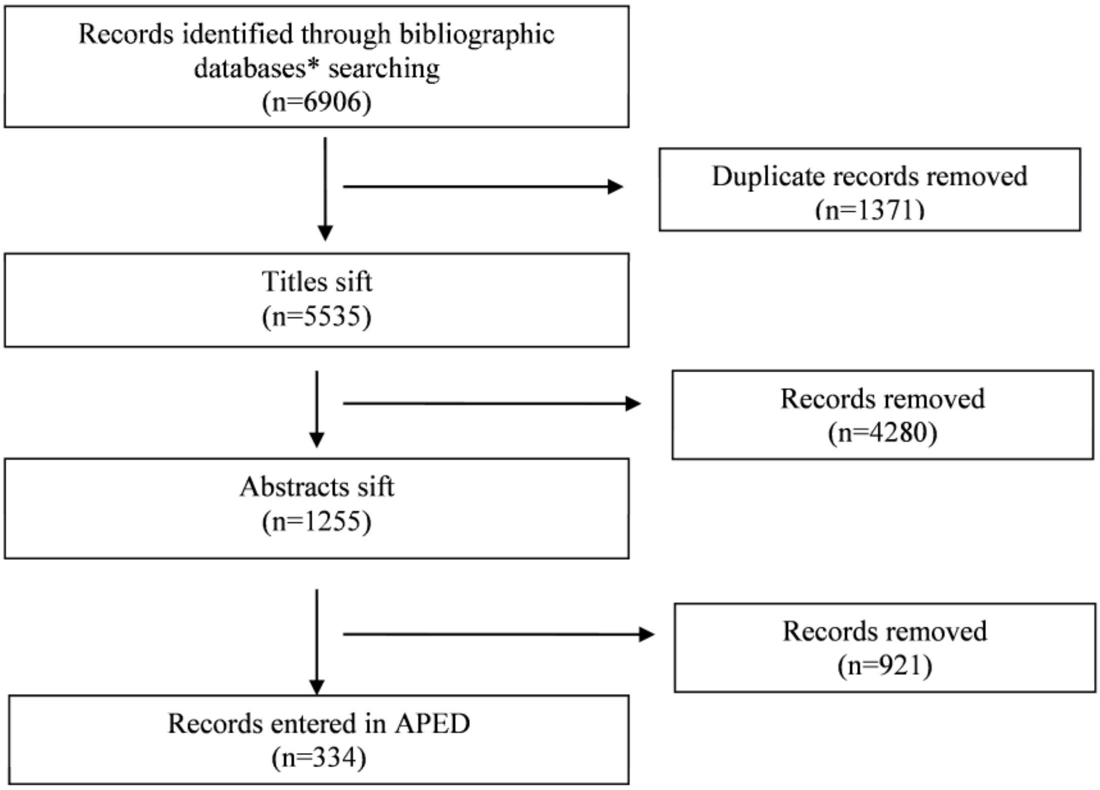

*Medline, Embase and Web of Science

First search performed in April 2009 (with no time limits), then updated in Jul 2010, Jul 2011, March 2013

Search strategy and search string to identify studies of traffic pollution and asthma prevalence

Medline, Embase and ISI Web of Science were searched from the default start date to 1 March 2103. Terms relating to exposure, health outcomes and study design were linked together using Boolean logic as follows:

(aerosol? OR air poll$ OR black smoke OR carbon monoxide OR nitrogen dioxide OR nitrogen oxides OR NO2 OR Nox OR O3 OR ozone OR particle? OR particulate? OR photochemical oxidant? OR SO2 OR sulphate OR sulphate OR sulfur dioxide OR sulphur dioxide)

AND

(asthma OR bronchitis OR bronchial hyperreactivity OR bronchial hyperresponsiveness OR cough OR dysp?nea OR FEV OR peak expiratory flow rate OR PEFR OR phlegm OR respirat$ OR ventilatory lung function OR wheez$)

AND

(cohort OR cross-sectional OR inciden$ OR multicenter OR multicity OR onset OR prevalen$ OR prospective OR questionnaire)

PRISMA Flow Diagram Stage 2

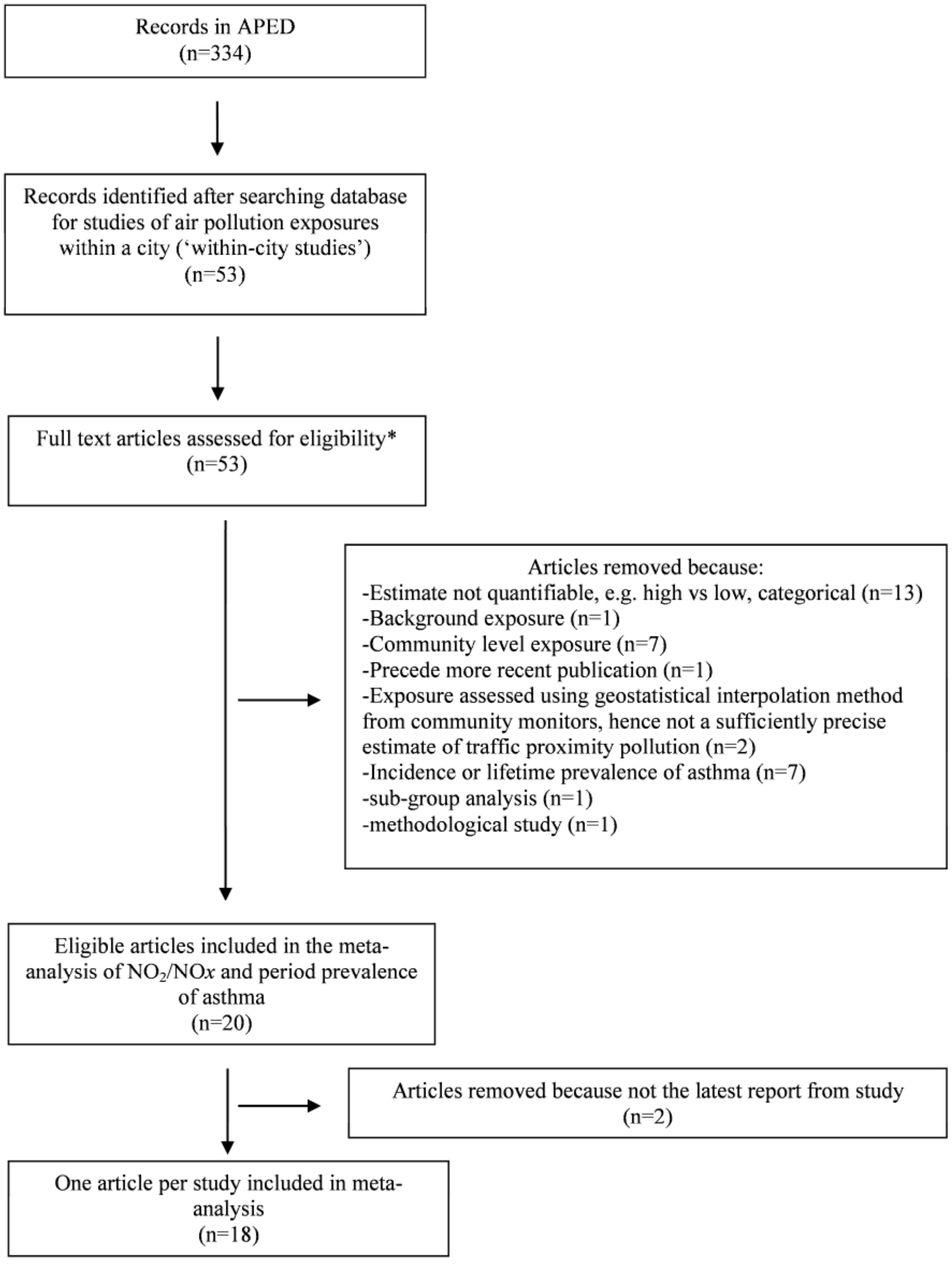

* Eligibility criteria: must be a within-community population based study with estimates that quantified in continuous form associations between NO_2 and prevalence of asthma, defined as 12 months period prevalence of asthma symptoms or asthma diagnosis
