# Supplementary material for: Traffic-related pollution and asthma prevalence in children. Quantification of associations with nitrogen dioxide
Source: Air Qual Atmos Health. 2014 May 10;7(4):459–66. doi: 10.1007/s11869-014-0265-8 (PMC4239711; doi:10.1007/s11869-014-0265-8)
Supplement: Supplementary file 2 — (PDF 60 kb) [file 11869_2014_265_MOESM2_ESM.pdf]

Supplementary material for *Traffic-related pollution and asthma prevalence in children. Quantification of associations with nitrogen dioxide*.

Online Resource 2

Details of eligible estimates for NO_2 and asthma prevalence from which the 18 estimates used in the meta-analysis were selected

access_id	rm_id	author	Study_name	Year	comparison	spatial	assessment	exposure	parameter_abbr	pollavg_yr	pollrange_yr	pollavgun	range	age	outcome	estimate	precision	period	followup	sex	method	type	unit	stand_or	stand_lci	outcategory	stand_se	stand_est	stand_uci	assess_id
144	594	Brauer, M.	PIAMA	2002	within community	home	LUR	NO2	PP	25.60	12.6-58.4	ug/m3	10.3	2	wheeze	1.13	0.99	1yr	n.a	F+M	Logistic regression	OR, LCI	ug/m3	1.13	0.99	wheeze	0.06552	0.118657896	1.28	6
147	594	Brauer, M.	PIAMA	2002	within community	home	LUR	NO2	PP	25.60	12.6-58.4	ug/m3	10.3	2	asthma, dx	1.18	0.93	1yr	n.a	F+M	Logistic regression	OR, LCI	ug/m3	1.17	0.93	asthma	0.11793	0.16069363	1.48	6
168	161	Brauer, M.	PIAMA	2007	within community	home	LUR	NO2	PP	25.20	12.6-58.4	ug/m3	10.6	4	wheeze	1.16	0.98	1yr	n.a	F+M	Logistic regression	OR, LCI	ug/m3	1.15	0.98	wheeze	0.08116	0.140018873	1.35	6
171	161	Brauer, M.	PIAMA	2007	within community	home	LUR	NO2	PP	25.20	12.6-58.4	ug/m3	10.6	4	asthma, dx	1.29	0.99	1yr	n.a	F+M	Logistic regression	OR, LCI	ug/m3	1.27	0.99	asthma	0.1274	0.240228508	1.63	6
871	8441	Esplugues, A.	INMA - Valencia	2011	within community	home	LUR	NO2	PP	27.40	18.7	ug/m3	10	1	wheeze	1.04	0.85	1yr	n.a	F+M	Logistic regression	OR, LCI	ug/m3	1.04	0.85	wheeze	0.10293	0.039220713	1.27	6
261	705	Gauderman, W.J.	CHS	2005	within community	home	Home study monitors	NO2	PP	30.80	12.9-51.5	ppb	5.7	14-17	wheeze, recent	1.72	1.07	1yr	n.a	F+M	Logistic regression	OR, LCI	ppb	1.64	1.06	wheeze	0.2221	0.497358141	2.54	14
262	705	Gauderman, W.J.	CHS	2005	within community	home	Home study monitors	NO2	PP	30.80	12.9-51.5	ppb	5.7	14-17	wheeze, recent with exercise	2.01	1.08	1yr	n.a	F+M	Logistic regression	OR, LCI	ppb	1.90	1.07	wheeze	0.29065	0.640249743	3.35	14
688	16	Gehring, U.	PIAMA	2010	within community	home	LUR	NO2	PP	25.40	12.6-58.4	ug/m3	10.4	8	asthma	1.22	0.94	1yr	8yr	F+M	Logistic regression	OR, LCI	ug/m3	1.21	0.94	asthma	0.12791	0.191202749	1.56	6
691	16	Gehring, U.	PIAMA	2010	within community	home	LUR	NO2	PP	25.40	12.6-58.4	ug/m3	10.4	8	wheeze	1.01	0.86	1yr	8yr	F+M	Logistic regression	OR, LCI	ug/m3	1.01	0.87	wheeze	0.07887	0.009567626	1.18	6
694	16	Gehring, U.	PIAMA	2010	within community	home	LUR	NO2	PP	25.40	12.6-58.4	ug/m3	10.4	8	asthma, sx	1.13	0.96	1yr	8yr	F+M	Logistic regression	OR, LCI	ug/m3	1.12	0.96	asthma	0.07998	0.117516955	1.32	6
873	8442	Gruzieva, O	BAMSE	2013	within community	home	Dispersion model	NOx	PP	11.80	46.8	ug/m3	46.8	12	wheeze	0.74	0.28	1yr	n.a	F+M	Logistic regression	OR, LCI	ug/m3	0.94	0.76	wheeze	0.10595	-0.064338695	1.15	2
872	8442	Gruzieva, O	BAMSE	2013	within community	home	Dispersion model	NOx	PP	11.80	46.8	ug/m3	46.8	12	asthma, dx	0.86	0.26	1yr	n.a	F+M	Logistic regression	OR, LCI	ug/m3	0.97	0.75	asthma	0.13041	-0.032227113	1.25	2
769	707	Hirsch, T.	Hirsch 1999	1999	within community	home	Interpolation model (kriging)	NO2	PP	33.80	17.1-56.0	ug/m3	10	5-7, 9-11	asthma, dx	1.16	0.94	1yr	n.a	F+M	Logistic regression	OR, LCI	ug/m3	1.16	0.94	asthma	0.10729	0.148420005	1.43	4
767	707	Hirsch, T.	Hirsch 1999	1999	within community	home	Interpolation model (kriging)	NO2	PP	33.80	17.1-56.0	ug/m3	10	5-7, 9-11	wheeze	1.13	0.93	1yr	n.a	F+M	Logistic regression	OR, LCI	ug/m3	1.13	0.93	wheeze	0.09938	0.122217633	1.37	4
401	25	Janssen, N.A.	Janssen 2003	2003	within community	school	School study monitors	NO2	PP	34.80	26.8-44.4	ug/m3	17.6	7-12	wheeze, current	1.74	0.99	1yr	n.a	F+M	Logistic regression	OR, LCI	ug/m3	1.37	0.99	wheeze	0.16348	0.314707451	1.89	15
746	442	Kim, J. J.	Kim 2004	2004	within community	school	School study monitors	NO	PP	25.00	11-38	ppb	11.6	7-10	asthma	1.05	0.98	1yr	n.a	F+M	Multilevel	OR, LCI	ppb	1.03	0.99	asthma	0.02432	0.033702313	1.08	15
745	442	Kim, J. J.	Kim 2004	2004	within community	school	School study monitors	NO2	PP	23.00	19-31	ppb	3.6	7-10	asthma	1.02	0.97	1yr	n.a	F+M	Multilevel	OR, LCI	ppb	1.03	0.96	asthma	0.03724	0.028754468	1.11	15
744	442	Kim, J. J.	Kim 2004	2004	within community	school	School study monitors	NOx	PP	49.00	33-69	ppb	14.9	7-10	asthma	1.04	0.97	1yr	n.a	F+M	Multilevel	OR, LCI	ppb	1.01	0.99	asthma	0.01247	0.013759867	1.04	15
858	21	Kim, J-L	Kim 2011	2011	within community	school	School study monitors	NO2	PP	30.70	16.5-48.6	ug/m3	10	10	asthma, current	0.99	0.79	1yr	n.a	F+M	Multilevel	OR, LCI	ug/m3	0.99	0.79	asthma	0.11514	-0.010050336	1.24	15
856	21	Kim, J-L	Kim 2011	2011	within community	school	School study monitors	NO2	PP	30.70	16.5-48.6	ug/m3	10	10	wheeze	1.27	1.06	1yr	n.a	F+M	Multilevel	OR, LCI	ug/m3	1.27	1.06	wheeze	0.09222	0.2390169	1.52	15
617	4734	Kramer, U.	GINIplus and LISAplus	2009	within community	home	LUR	NO2	PP	23.70	13.6-42.1	ug/m3	9	6	a/s/o BR/Asthma, sx	0.64	0.4	1yr	n.a	F+M	Cox regression	RR, LCI	ug/m3	0.61	0.36	asthma	0.26644	-0.495874558	1.03	6
614	4734	Kramer, U.	GINIplus and LISAplus	2009	within community	home	LUR	NO2	PP	23.70	13.6-42.1	ug/m3	9	6	a/s/o BR/Asthma, dx	0.95	0.59	1yr	n.a	F+M	Cox regression	RR, LCI	ug/m3	0.94	0.56	asthma	0.27003	-0.056992549	1.60	6
730	710	Kramer, U.	Dusseldorf study	2000	within community	home	Interpolation model (other)	NO2 outdoors	PP	54.20	43.0-67.5	ug/m3	10	9	wheeze	1.70	0.9	1yr	n.a	F+M	Logistic regression	OR, LCI	ug/m3	1.70	0.90	wheeze	0.32448	0.530628251	3.21	5
731	710	Kramer, U.	Dusseldorf study	2000	within community	personal	Interpolation model (other)	NO2 personal	PP	25.40	11.1-69.9	ug/m3	10	9	wheeze	0.54	0.22	1yr	n.a	F+M	Logistic regression	OR, LCI	ug/m3	0.54	0.22	wheeze	0.45813	-0.616186139	1.33	5
131	419	Mi, Y.H.	MI 2006	2006	within community	school	School study monitors	NO2	PP	63.00	47-83	ug/m3	10	13 avg	wheeze, current	1.00	0.74	1yr	n.a	F+M	Logistic regression	OR, LCI	ug/m3	1.00	0.74	wheeze	0.15363	0	1.35	15
133	419	Mi, Y.H.	MI 2006	2006	within community	school	School study monitors	NO2	PP	53.00	47-83	ug/m3	10	13 avg	asthma, current	1.44	1.06	1yr	n.a	F+M	Logistic regression	OR, LCI	ug/m3	1.44	1.06	asthma	0.15631	0.364643114	1.96	15
232	1	Morgenstern, V.	TRACPA	2008	within community	home	LUR	NO2	PP	34.60	16.0-73.7	ug/m3	6.4	6	asthma or a/o/s BR, sx	1.03	0.9	1yr	n.a	F+M	Logistic regression	OR, LCI	ug/m3	1.05	0.85	asthma	0.10756	0.046185629	1.29	6
223	1	Morgenstern, V.	TRACPA	2008	within community	home	LUR	NO2	PP	34.60	16.0-73.7	ug/m3	6.4	6	asthma or a/o/s BR, dx	1.04	0.67	1yr	n.a	F+M	Logistic regression	OR, LCI	ug/m3	1.06	0.53	asthma	0.35052	0.061282364	2.11	6
256	8427	Oftedal, B.	Oslo Birth Cohort	2009	within community	home	Dispersion model	NO2 previous year	PP	25.20	1.4-65.1	ug/m3	17.9	9-10	wheeze, current severe	1.10	0.79	1yr	n.a	F+M	Logistic regression	OR, LCI	ug/m3	1.05	0.88	wheeze	0.09435	0.053245911	1.27	2
254	8427	Oftedal, B.	Oslo Birth Cohort	2009	within community	home	Dispersion model	NO2 previous year	PP	25.20	1.4-65.1	ug/m3	17.9	9-10	wheeze, current	1.01	0.83	1yr	n.a	F+M	Logistic regression	OR, LCI	ug/m3	1.01	0.90	wheeze	0.05595	0.005558844	1.12	2
819	87	Penard-Morand C	The French 6 Cities Study	2010	within community	school	Dispersion model	NO2	PP	43.65	18.5	ug/m3	18.5	9-11	asthma in the last yr	1.37	0.85	1yr	n.a	F+M	Logistic regression	OR, LCI	ug/m3	1.19	0.92	asthma	0.13164	0.170167967	1.53	2
814	87	Penard-Morand C	The French 6 Cities Study	2010	within community	school	Dispersion model	NO2	PP	43.65	18.5	ug/m3	18.5	9-11	asthma, exercise induced	1.05	0.72	1yr	n.a	F+M	Logistic regression	OR, LCI	ug/m3	1.03	0.84	asthma	0.10405	0.026373062	1.26	2
820	87	Penard-Morand C	The French 6 Cities Study	2010	within community	school	Dispersion model	NOx	PP	78.78	52.1	ug/m3	52.1	9-11	asthma in the last yr	1.32	1	1yr	n.a	F+M	Logistic regression	OR, LCI	ug/m3	1.05	1.00	asthma	0.02719	0.053288241	1.11	2
815	87	Penard-Morand C	The French 6 Cities Study	2010	within community	school	Dispersion model	NOx	PP	78.78	52.1	ug/m3	52.1	9-11	asthma, exercise induced	1.25	1.11	1yr	n.a	F+M	Logistic regression	OR, LCI	ug/m3	1.04	1.02	asthma	0.01163	0.042829856	1.07	2
830	2072	Pikhart, H.	SAVIAH	2000	within community	home/school	LUR	NO2	PP	35.80	27.9-45.3	ug/m3	10	7-10	wheeze or whistling in the chest	1.16	0.97	1yr	n.a	F+M	Logistic regression	OR, LCI	ug/m3	1.16	0.97	wheeze	0.09126	0.148420005	1.39	6
877	8444	Sonnenschein-van der Voort, A. M.	Generation R Study	2012	within community	home	Dispersion model	NO2	PP	36.22	4.28	ug/m3	10	3	wheeze	0.97	0.72	1yr	n.a	F+M	Logistic regression	OR, LCI	ug/m3	0.97	0.72	wheeze	0.15206	-0.030459207	1.31	2
881	8445	Svendsen, E. R.	El Paso	2012	within community	home/school	LUR	NO2 valley/ upland	PP	23.30		ppb	10	10	wheeze	0.90	0.67	1yr	n.a	F+M	Logistic regression	OR, LCI	ppb	0.90	0.67	wheeze	0.15057	-0.105360516	1.21	6
879	8445	Svendsen, E. R.	El Paso	2012	within community	home/school	LUR	NO2 valley/ upland	PP	23.30	9.1	ppb	10	10	asthma, current - physician dx	1.12	0.84	1yr	n.a	F+M	Logistic regression	OR, LCI	ppb	1.12	0.84	asthma	0.14678	0.113328685	1.49	6
766	1581	Zhao, Z.	Zhao 2008	2008	within community	school	School study monitors	NO2	PP	52.30	37.9-65.2	ug/m3	10	11-15	wheeze or whistling in the chest	1.00	0.83	1yr	n.a	F+M	Logistic regression	OR, LCI	ug/m3	1.00	0.83	wheeze	0.09507	0	1.20	15
