# Supplementary material for: Traffic-related pollution and asthma prevalence in children. Quantification of associations with nitrogen dioxide
Source: Air Qual Atmos Health. 2014 May 10;7(4):459–66. doi: 10.1007/s11869-014-0265-8 (PMC4239711; doi:10.1007/s11869-014-0265-8)
Supplement: Supplementary file 3 — (PDF 50 kb) [file 11869_2014_265_MOESM3_ESM.pdf]

Supplementary material for *Traffic-related pollution and asthma prevalence in children. Quantification of associations with nitrogen dioxide*.

Online Resource 3

Funnel plot of estimates for NO_2 (per 10µg/m^3) and asthma prevalence included in meta-analysis

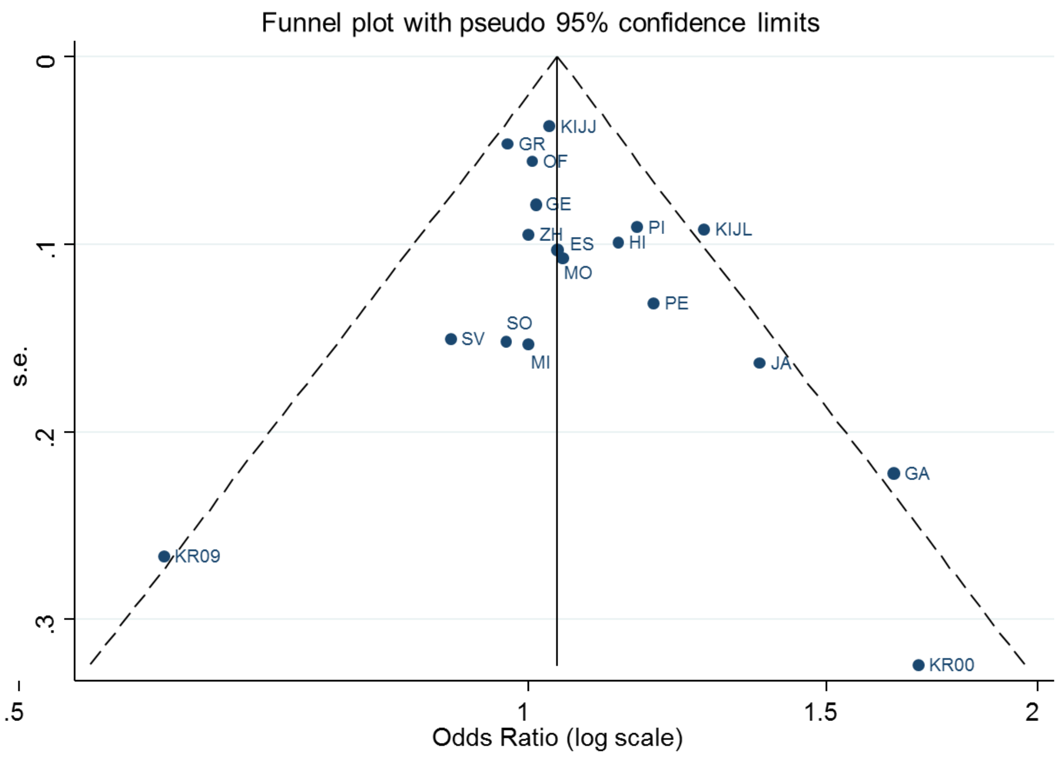

ES: Esplugues 2011, Spain

GA: Gauderman 2005, USA

GE: Gehring 2010, The Netherlands

GR: Gruzieva 2013, Sweden

HI: Hirsch 1999, Germany

JA: Janssen 2003, The Netherlands

KIJJ: Kim JJ 2004, USA

KIJL: Kim JL 2011, South Korea

KR00: Kramer 2000, Germany

KR09: Kramer 2009, Germany

MI: 2006, China

MO: Morgenstern 2008, Germany

OF: Oftedal 2009, Norway

PE: Penard-Morand 2010, France

PI: Pikhart 2000, Czech Republic

SO: Sonnenschein 2012, The Netherlands

SV: Svendsen 2012, USA

ZH: Zhao 2008, China
